# The Role of Calcium in Wound Healing

**DOI:** 10.3390/ijms22126486

**Published:** 2021-06-17

**Authors:** Thayaalini Subramaniam, Mh Busra Fauzi, Yogeswaran Lokanathan, Jia Xian Law

**Affiliations:** Centre for Tissue Engineering and Regenerative Medicine, Universiti Kebangsaan Malaysia Medical Centre, Cheras, Kuala Lumpur 56000, Malaysia; subramaniamthayaalini@gmail.com (T.S.); fauzibusra@ukm.edu.my (M.B.F.); lyoges@ppukm.ukm.edu.my (Y.L.)

**Keywords:** calcium, skin, wound healing, fibroblast, keratinocyte

## Abstract

Skin injury is quite common, and the wound healing is a complex process involving many types of cells, the extracellular matrix, and soluble mediators. Cell differentiation, migration, and proliferation are essential in restoring the integrity of the injured tissue. Despite the advances in science and technology, we have yet to find the ideal dressing that can support the healing of cutaneous wounds effectively, particularly for difficult-to-heal chronic wounds such as diabetic foot ulcers, bed sores, and venous ulcers. Hence, there is a need to identify and incorporate new ideas and methods to design a more effective dressing that not only can expedite wound healing but also can reduce scarring. Calcium has been identified to influence the wound healing process. This review explores the functions and roles of calcium in skin regeneration and reconstruction during would healing. Furthermore, this review also investigates the possibility of incorporating calcium into scaffolds and examines how it modulates cutaneous wound healing. In summary, the preliminary findings are promising. However, some challenges remain to be addressed before calcium can be used for cutaneous wound healing in clinical settings.

## 1. Introduction

Wound healing is a complex process aimed at restoring the damaged skin to preserve tissue homeostasis. It involves the interaction between different cell types, growth hormones, cytokines, and a stable supply of metal ions, such as calcium, zinc, and magnesium [1]. Hemostasis, inflammation, proliferation, and remodeling are the four overlapping phases of normal wound healing [2,3]. When a tissue injury occurs, the coagulation pathway is triggered, resulting in the formation of a temporary fibrin matrix that allows cells to migrate to the wounded site. At the same time, platelet-derived factors attract leukocytes, thus activating the inflammatory response [4]. Then, platelets and immune cells secrete growth factors and cytokines, which promote wound re-epithelialization, extracellular matrix (ECM) deposition, and angiogenesis. Disruption to the normal wound healing process leads to the formation of a chronic wound that is much more difficult to heal [5].

Apart from being a critical coagulation factor during hemostasis, the calcium ion has also been shown to act as a fundamental cue, directing the cellular functions of different types of cells during wound healing. Calcium plays a vital role as the extracellular signaling molecule and intracellular second messenger for keratinocytes and fibroblasts. Previous studies have explored the impact of calcium concentrations on keratinocyte proliferation and differentiation [6,7]. However, the effects of calcium on dermal fibroblasts have yet to be fully elucidated. A modest number of studies have shown that calcium influences the morphology, proliferation, and collagen deposition of fibroblasts [8,9].

This review examines in depth the advances and emerging strategies using calcium to regulate cutaneous wound healing. In addition, this review also investigates the possibility of incorporating calcium into scaffolds to promote wound healing.

## 2. Skin Injury

As the first protective barrier of the human body, skin is subjected to various environmental insults from time to time [10,11]. In addition, pathological changes such as diabetes mellitus and reduced blood circulation can also lead to disruptions in skin integrity [12]. A wound is defined as a break in the skin’s epithelial integrity, which may affect the structure and function of the underlying tissue. The severity of skin injuries such as burns can be classified based on the depth of the injuries. Generally, the deeper and the larger the area affected, the more serious the injury.

### Prevalence of Skin Injury and Medical Cost

Wound management is a major public health issue worldwide. It is anticipated that the prevalence of chronic wounds in developed countries is approximately 1–2% of the total population [13]. In a study, the authors reported that around 15% of the 8.2 million Medicare beneficiaries in the year 2014 had a skin wound or infection and a total of USD 28.1 billion to USD 96.8 billion was spent on wound management [14].

In developing countries like Malaysia, diabetic wounds seem to have higher prevalence [15]. It is estimated that 2.6 million Malaysians, or 15.2% of the total population, are suffering from diabetes. It is more alarming that this number is predicted to increase dramatically in the coming years [16]. Diabetic foot ulcer (DFU) affects 15–25% of diabetic patients during their lifetime and it is also the leading cause of amputation. This major diabetic complication affects not only patients, but also their families and the economy. The management of DFU will inevitably increase the medical expenses of the healthcare system [17]. Sultanah et al. found that various care modalities, i.e., antibiotics, surgical debridement, and dressing, should be used concurrently to treat DFU [18]. A routine post-discharge follow-up will also guarantee the patient’s commitment to prescription and dressing procedures, thus reducing the occurrence of complications.

The medical cost for wound treatment is high and continues to grow rapidly. Hence, there is a need for researchers, scientists, doctors, and the government to work closely in providing an effective wound care scheme to ease the financial burden of patients and the country.

## 3. Four Stages of Wound Healing

**Figure 1:**
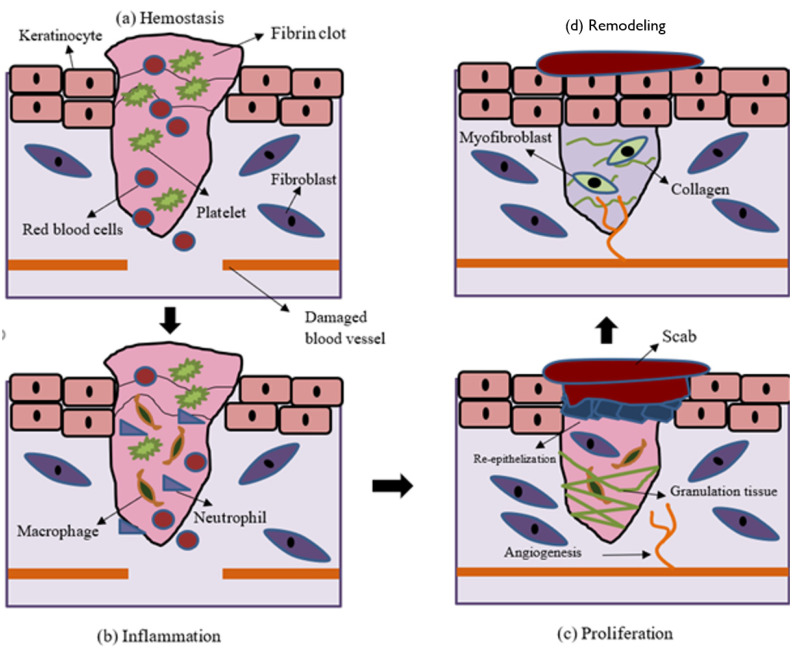
Illustration of the wound-healing process. Wound healing is divided into four phases: (**a**) hemostasis, (**b**) inflammation, (**c**) proliferation, and (**d**) remodeling. Hemostasis involves the formation of fibrin clot to stop the bleeding. Inflammation phase is associated with the infiltration of leukocytes to remove the cellular debris and infectious agents. During the proliferation phase, keratinocytes, fibroblasts, and endothelial cells undergo proliferation and migration to re-epithelialize, lay down collagen matrix, and revascularize the wound, respectively. Finally, the wound become mature during the remodeling phase.

Hemostasis, inflammation, proliferation, and tissue remodeling are the four basic processes of wound healing [19]. Basically, acute wounds tend to heal faster and with less complications than chronic wounds. Cutaneous wound reconstruction occurs to preserve the integrity of the damaged tissue and recapitulates embryonic skin growth in many ways. Both mechanisms include differentiation, migration, proliferation, and apoptosis of different types of cells to create the multi-layered skin tissue [20]. Cell and biochemical processes in wound healing are explained in brief in the sub-sections below and depicted in Figure 1 [21].

### 3.1. Hemostasis

Upon injury, blood vessels contract to deter blood leakage, followed by primary and secondary hemostasis. Primary hemostasis involves platelet aggregation at the injury site through interaction with the ECM protein components, such as fibronectin and collagen. Secondary hemostasis activates the coagulation pathway, which transforms fibrinogen into fibrin mesh at the injury site to capture the red blood cells, thereby stopping the bleeding.

### 3.2. Inflammation

The inflammatory phase typically lasts a few days and involves leukocyte chemotaxis to facilitate the removal of cellular debris and pathogen. In the beginning, neutrophils are more abundant, but are subsequently outnumbered by macrophages. Their functions are to phagocytose the cellular debris and pathogens. At the same time, the recruited white blood cells (WBCs) and thrombocytes intensify the inflammation cycle by releasing more mediators and cytokines. In addition, WBCs also secrete PDGF and TGF-B, which stimulate migration, proliferation, and differentiation of fibroblasts, which are responsible for collagen synthesis.

### 3.3. Proliferation

This phase is characterized by the development of granulation tissue, re-epithelialization, and neovascularization. These processes may take up to several weeks. Neovascularization happens through both angiogenesis, which involves the development of new blood vessels from existing arteries, and vasculogenesis, which is characterized by the formation of new arteries by endothelial progenitor cells (EPCs). Neovascularization restores the nutrient supply to the tissue. Re-epithelialization starts with the migration of keratinocytes from the perimeter of the wound and gradually reduces the wound size. Originally, only a thin, shallow layer of epithelial cells is laid down. Over time, a thicker and more robust layer of cells covers the wound. Macrophages acquire the M2 phenotype and secrete anti-inflammatory mediators that stimulate collagen deposition, fibroblast proliferation, and angiogenesis. The wound begins to mature when the provisional fibrin matrix is replaced with collagen fibers.

### 3.4. Remodeling

Wound remodeling is a long process that can last for years. It involves the degradation of excessive collagen and the beginning of wound contraction. After healing, the scar tissue will never regain full strength, instead only achieving approximately 80% of the original tensile strength.

## 4. Current Treatments for Skin Injury

Disruptions in epidermal and dermal layer integrity can lead to many serious consequences, including death. Rapid healing is vital to reduce morbidity and mortality [22]. The debridement or elimination of non-viable tissue is an important step in wound treatment. Surgical or autolytic/enzymatic approaches may be used to do this. The goal of debridement is to remove the necrotic tissue that is prone to infection and inhibit wound healing, as well as to expose the stable, well-perfused tissue that is capable of supporting the migration and proliferation of epithelial cells to repopulate the wound bed. Many wound dressings have been developed both to shield the wound from infection and to facilitate wound healing. Occlusive dressing promotes angiogenesis and re-epithelization by creating a hypoxic and moist wound environment, respectively [23,24]. The presence of a small amount of exudate promotes autolytic debridement, which facilitates wound healing [25]. Table 1 shows the pros and cons of different types of commercially available dressings used to promote wound healing.

## 5. Calcium and Wound Healing

Calcium ions are known to regulate the intracellular signals that modulate many cellular activities. As early as 1983, Chapman demonstrated changes in the calcium gradient after chemical or physical stimuli, which coincided with the activation of calcium channels around the cell membrane [31]. Local calcium has been shown to modulate the proliferation, differentiation, and maturation of keratinocytes and fibroblasts, as well as the formation of epidermal lipid barrier function via signal transduction and gene expression [32].

Xu and Chisholm examined the early stages of wound healing in a living organism, i.e., the worm *Caenorhabditis* *elegans*, expressing the fluorescent calcium sensor. They discovered that both laser and mechanical wounding were effective in stimulating the formation of a rapid calcium wave that spread from the site of injury and contributed to a sustained rise in epidermal calcium. Further analysis showed that this calcium flash depended on the TRP channel TRPM (GTL-2 in worms). The results from this study indicated that calcium was involved in the earliest wound signaling activities and may play a significant role in modulating would healing [33]. Furthermore calcium flux has been shown to promote wound healing in early *Xenopus* embryos and a rapid, transient increase in intracellular calcium has been observed in the in vitro ‘scratch’ wound assay, as well as the single-cell wound healing assay [34,35].

In a recent study, the electrostatic interaction of gelatin and alginate, the incorporation of cellulose nanocrystals, and the cross-linking of alginate with calcium ions were used to create the SA/Ge/CNC scaffold [36]. In the final step, calcium ions were used to crosslink the G-blocks in the alginate molecules and form the egg-box structure, creating the second network of the scaffold. The results showed that cells were able to effectively attach and spread on the scaffold, confirming its good cytocompatibility. The SA/Ge/CNC scaffolds demonstrated great efficacy in speeding up wound healing in an in vivo wound healing model. When all these factors are considered, the SA/Ge/CNC scaffold, with good porosity, moderate water absorption capacity, outstanding mechanical characteristics, and excellent biocompatibility, is a promising option for skin tissue engineering.

The results of these experiments demonstrated the importance of determining the function of calcium in cutaneous wound healing and its mechanism of action. This information will contribute to better wound management to achieve rapid wound closure and to restore the original functions and mechanical strength of the regenerated tissue.

### 5.1. Effects of Calcium on Keratinocytes

Calcium is the main regulator of keratinocyte differentiation [37]. A calcium gradient within the epidermis facilitates the differentiation of keratinocytes as they cross various strata, eventually forming the semipermeable stratum corneum [38]. The stratum basale and spinosum have lower calcium concentrations, and the calcium concentration gradually increases towards the stratum granulosum and decreases again in the stratum corneum. Calcium is essential for keratinocyte differentiation, whereby it stimulates the differentiation of basal keratinocytes in the stratum basale and spinosum, as well as triggering terminal differentiation of cells in the stratum granulosum. Calcium is known to regulate the expression of keratinocyte differentiation-specific genes, such as transglutaminase, involucrin, loricrin, cytokeratin 1, cytokeratin 10, and filaggrin [39].

In an in vitro study, researchers studied the influence of calcium concentrations, i.e., 1.4 mM, 0.4 mM, and 0.03 mM, on the proliferation and differentiation of primary keratinocytes [40]. The results showed that cell viability and proliferation were inhibited at higher calcium concentrations (1.4 mM and 0.4 mM). In another study, the authors found that keratinocytes cultured in lower calcium concentrations (0.05–0.1 mM) proliferated steadily but were unable to differentiate and form stratified layers [38]. The keratinocytes begin to differentiate and establish intracellular mechanisms that are essential for differentiation once the calcium concentration was increased. Calcium is known to regulate the formation of desmosomes, adherens junctions, and tight junctions, as well as activating the calcium-sensing receptor (CaSR), which is required to initiate the intracellular mechanisms that regulate keratinocyte differentiation and survival [39,41,42].

### 5.2. Effects of Calcium on Fibroblasts

Fibroblasts also respond to extracellular calcium but are 100 times less sensitive to it than keratinocytes. Fibroblasts mainly use calcium intracellularly for contraction and this contraction is important in reducing the wound size during wound healing [43]. In another study, the authors found that intracellular calcium is needed for cell–cell adhesion in fibroblasts by mediate remodeling of actin and the recruitment of cadherins into the intracellular junctions [44].

The response of fibroblasts to extracellular calcium is not as widely studied as their response to intracellular calcium. Nonetheless, extracellular calcium has been found to affect the wound healing process and may be used as a novel biomolecule to modulate skin wound healing. Navarro-Requena et al. conducted a study to examine the effects of extracellular calcium on skin fibroblasts cultured in vitro [8]. Supplementation of extracellular calcium was found to increase the cell metabolic activity, migration, MMP production, collagen synthesis, and cytokine release, as well as decreasing the cell contraction ability. Interestingly, the changes in migration and contraction ability were found to be dose-dependent. Kawai et al. also investigated the use of calcium to facilitate cutaneous wound healing [9]. The study discovered that intravenous injection of calcium-based nanoparticles (NPs) and calcium chloride accelerated wound healing but only calcium chloride was able to hasten wound healing when they were applied topically. The topical application of calcium-based NPs failed to promote wound healing probably due to the failure of calcium release from the NPs. NPs delivered intravenously accumulated at the wound site and improved the calcium absorption of fibroblasts, subsequently hastening wound healing by enhancing fibroblast proliferation and contraction.

### 5.3. Effects of Calcium on Angiogenesis

Calcium is a key signaling molecule for a variety of signaling pathways that regulate angiogenesis [45]. The majority of the mitogens, including the angiogenic factors, are known to activate calcium influx through the opening of plasma membrane calcium channels or releases from intracellular organelles such as endoplasmic reticulum [46]. Calcium influx into endothelial cells has been reported to play a crucial role in endothelial cell migration, adhesion, proliferation, and vessel formation in vitro and in vivo [47,48]. Blockage of non-voltage gated calcium channels by carboxyamidotriazole was found to inhibit the above cellular processes [48].

A recent study tested the anti-infective and wound healing abilities of protamine nanoparticles (NPs)/hyaluronan oligosaccharide calcium alginate (HAO CaAlg) hydrogel to treat wounds created in a type I diabetic rat model. The results showed that protamine NPs/HAO hydrogel showed better bactericidal activity and accelerated wound healing by encouraging angiogenesis and improving exudate absorption. Better exudate absorption helped to keep the wound moist and aided in the removal of foreign substances. Furthermore, the hydrogel allowed the quicker release of protamine NPs and HAO at pH 8.0, a pH which mimics the diabetic wound microenvironment, to improve its antibacterial action and angiogenesis effect [49]. All these findings suggest that calcium could be used to modulate wound angiogenesis, eventually accelerating the healing process.

### 5.4. Role of Calcium in Wound Healing

The importance of calcium in wound healing is evident in the delayed wound healing and higher prevalence of chronic wound formation in animals with dietary calcium deficiency and the presence of calcium chelating agents in their diet [50,51]. The calcium concentration in the wound area varies in accordance with the biochemical activities of the healing process. The extracellular calcium concentration has been shown to increase upon injury, persisting through the inflammatory and proliferative phases, and declining during the remodeling phase [52].

During the hemostasis phase, calcium aids in blood clotting by facilitating the formation of the platelet plug [53].

In the inflammatory phase, high extracellular calcium is thought to enter neutrophils and cause the intracellular calcium to increase, subsequently modulating the neutrophil function [54]. One of the hallmarks of the proliferative phase is the resurfacing of the wound with a new epithelium [26]. Extracellular calcium is a key regulator of epidermal homeostasis and its receptor (CaSR) sends calcium signals to promote keratinocyte adhesion, differentiation, and survival by inducing intracellular calcium and E-cadherin-mediated signaling [42]. The rapid induction of calcium ion propagation at the wound site signifies a transcription-independent damage signal to initiate epithelial healing [55].

Calcium ions, also known as clotting factor IV, can trigger the intrinsic coagulation cascade along with other clotting factors, thus accelerating the synthesis of enough thrombin to facilitate early fibrin formation [56,57]. Calcium ions mediate the binding of the tenase and prothrombinase complexes to the phospholipid surfaces expressed by platelets to the procoagulant microparticles or microvesicles secreted by them and are required for stable platelet incorporation into the developing thrombus [58].

Keratinocyte proliferation was found to be inversely proportional to the extracellular calcium concentration, whereby the cell proliferation was faster at a low calcium concentration and cells became differentiated at higher calcium concentrations [59]. Tu et al. reported that wound-induced calcium ion propagation is essential for successful keratinocyte migration [60,61]. The strongest response was found in the stratum basale, where CaSR was strongly expressed, and injury induced the cells to proliferate and migrate. Endogenous CaSR stimulation improved wound re-epithelization by increasing calcium ion signals and E-cadherin membrane expression. These results showed that CaSR is vital in epidermal regeneration, and it has great potential as a therapeutic target to accelerate wound healing [62].

Excessive calcium concentrations in the wound area inhibited keratinocyte proliferation and migration and is thought to slow down wound healing [63,64]. However, an appropriate elevation of the calcium concentration in the wound could be beneficial, as keratinocytes have been shown to migrate towards a high calcium concentration through galvanotaxis studies [65,66]. Therefore, it is extremely important to determine the appropriate calcium concentration that would facilitate epidermal cell proliferation and migration in vivo, which could be a key factor in facilitating the process of re-epithelization and ultimately wound healing [67]. Less is known about the effects of changes in the calcium concentration during wound healing on fibroblasts and endothelial cells. However, it has been postulated that a higher calcium concentration in the wounds could increase collagen synthesis and blood vessel formation [68]. Finally, in the remodeling phase, the epidermal hyperplasia is diminished, and the dermal collagen is reorganized. However, the role of calcium in these tissue events remains unclear.

## 6. Calcium-Releasing Scaffolds

The functionalization of scaffolds that have been traditionally designed to provide a physical barrier, mechanical strength, and excellent biocompatibility to support cell bioactivities, and to maintain a moist wound environment, is a new trend in the field of wound healing [69]. Many biomolecules can be incorporated into these scaffolds to functionalize them and calcium is one of the relatively new biomolecules that has been added to facilitate wound healing. Calcium has been chosen because it plays an important role in normal skin physiology and wound healing.

### 6.1. Dressings with Calcium Ions

Dressings are used to protect wounds and help to maintain a moist wound environment, which favors wound healing. Particulate leaching, freeze-drying, supercritical fluid technology, thermally mediated phase separation, quick prototyping, powder compaction, sol-gel, melt molding, and electrospinning are some the techniques used to fabricate wound dressings [70]. Many forms of wound dressing have been developed and used in the clinic. However, preventing wound infection and hastening the regeneration of chronic wounds remain challenges when using existing dressings.

Several calcium-containing dressings have been identified to facilitate wound healing. Wang et al. treated wounds created on a diabetic rat model with calcium alginate dressing [70,71]. Their results showed that the calcium alginate dressing expedited wound healing compared to the Vaseline dressing by enhancing the synthesis of collagen type I and wound re-epithelialization, as well as suppressing the inflammation. Zhao et al. prepared chitosan-calcium alginate dressing and used it to promote wound healing in rats [72]. The dressing was able to reduce inflammation and enhance angiogenesis, leading to faster wound healing.

In another study, the authors prepared chitosan/gelatin/nanocrystalline cellulose/calcium peroxide films for wound treatment application [73]. Calcium peroxide is an oxygen releasing biomaterial and has been found to possess antibacterial properties. However, no in vitro and in vivo wound healing experiments were conducted by the authors. Jeong et al. used an in situ precipitation technique to produce calcium fluoride-containing composite hydrogel dressings and found that the presence of calcium fluoride enhanced the migration of fibroblasts and endothelial cells, and inhibited bacterial growth [73]. In vivo, a calcium fluoride composite hydrogel hastened wound healing by increasing the chemotaxis of immune cells, promoting the migration of surrounding skin cells, and accelerating the re-epithelialization process. The key findings from the above studies are summarized in Table 2. The findings of these studies clearly show that dressings with calcium can promote wound healing.

### 6.2. Calcium-Releasing Nanoparticles

NPs are nanometer-scale small structures which can bind and deliver ions, proteins, and other organic molecules. These tiny NPs can regulate the concentration of extracellular calcium by controlling the ion release, thus modulating wound healing. Table 3 summarizes the findings of papers using calcium-releasing NPs to promote wound healing.

In a study, the authors investigated the possibility of using a silver-exchanged calcium-doped ordered mesoporous silica sphere (AgCaMSS) as a hemostat. The mesoporous silica spheres (MSSs) were doped with calcium ions, which have been demonstrated to function as coagulation factor IV in the blood coagulation cascade to reduce hemorrhaging. The AgCaMSSs were found to be more effective compared to calcium-free MSSs in accelerating thrombosis and platelet adhesion in vitro and stopping bleeding in vivo [58]. In another study, the authors utilized the calcium ions released from wollastonite, a calcium silicate (CaSiO_3_), to promote hemostasis and wound healing. The wollastonite particles were doped with zinc to increase their antimicrobial activity. The zinc-doped wollastonite particles were found to be non-toxic to the fibroblasts at low concentrations (≤0.1 mg/mL), hastening the wound healing, and were antibacterial in vitro [67].

Navarro-Requena et al. prepared calcium-phosphate-based ormoglass NPs, coded SG5, and tested them on skin fibroblasts [8]. The calcium-releasing NPs were found to increase cell migration, collagen synthesis, and cytokine release, as well as decreasing the cell contraction ability compared the low-calcium group. Perez-Amodio et al. used electrospinning to prepare poly(lactic acid) (PLA) nanofiber mats with SG5 nanoparticles. The PLA-SG5 mats were found to accelerate the healing of pressure ulcers compared to the PLA mats and Mepilex^®^ as indicated by faster wound re-epithelialization, higher migration of fibroblasts from the surrounding tissue, higher collagen synthesis, and higher vascularity in diabetic mice with ischemic wounds. These results indicate that calcium-releasing NPs are beneficial for wound healing.

### 6.3. Engineered Skin with Calcium ions

Several studies have fabricated engineered skin using a scaffold loaded with calcium to enhance its wound healing ability. The key findings of these studies are summarized in Table 4. Fibrin is one of the biomaterials that has been used to prepare engineered skin. Calcium and/or thrombin can be used to crosslink the fibrinogen. Idrus et al. developed a bilayer skin consisting of a fibrin-fibroblast layer overlaid with a fibrin-keratinocyte layer to treat full-thickness wounds [12,76]. A concentration of 25 mM calcium chloride was used to facilitate fibrinogen polymerization. However, keratinocytes tend to differentiate when the calcium concentration rose above 0.1 mM [39]. Thus, the influence of calcium concentration on the keratinocyte entrapped within the fibrin scaffold was investigated. Keratinocytes were embedded within the fibrin hydrogel polymerized with 5, 10, and 25 mM calcium chloride [37]. Results showed that keratinocytes were not differentiated even at the highest calcium concentration. The authors postulated that this is due to the low concentration of extracellular calcium that can influx into the keratinocytes to activate the signaling pathways that regulate keratinocyte differentiation. Fibrin-based bilayered skins have been found to promote the healing of full-thickness wounds in mice and sheep [76].

In another study, the authors utilized the advantage of the calcium release from alginate-gelatin porous hydrogel scaffolds to promote blood clotting during the earliest phase of wound healing. The scaffolds were prepared using the freeze-gelation method and had suitable porosity, pore size, and mechanical properties, as well as excellent biocompatibility for skin tissue engineering. The application of these scaffolds with and without murine fibroblasts was found to promote the healing of second-degree burn wounds created on rats, with better results observed in the scaffolds with cells [56].

Ninan et al. prepared pectin/carboxymethyl cellulose/microfibrillated cellulose (pectin/CMC/MFC) composite scaffolds gelated with calcium ions for skin tissue engineering. The results showed that the scaffold with 0.1% (*w*/*v*) MFC has the highest biocompatibility. The authors postulated that the poor cell viability in other groups was due to excessive calcium efflux to the culture medium [68].

In a recent study, the authors prepared atorvastatin-loaded chitosan-hydroxyapatite composite scaffolds crosslinked with calcium chloride and found that the scaffolds demonstrated antibacterial activity and were effective in promoting wound healing in vivo [70]. The results from the above studies justify the incorporation of calcium into engineered scaffolds for skin tissue engineering purposes and further explored with Table 4.

## 7. Conclusions and Future Perspectives

A lot of achievements have been accomplished with the aim of providing better management of cutaneous wounds in the last two decades, but there is still more to be done. A crucial approach to ensuring better overall performance is the commitment to performing high-quality research and exploring new and niche areas, such as the incorporation of calcium into the treatment of cutaneous wounds.

Although studies have been performed on the role of calcium in wound healing, the effect of extracellular calcium in acute and chronic wound healing remains unclear. Since dermal fibroblasts and epidermal keratinocytes play an important role in the healing process, from the formation of granulation tissue to wound re-epithelialization and eventual wound remodeling, it is important to understand the effects of calcium concentration on these cells. Earlier data showed the potential of adding calcium into functional dressings to hasten cutaneous wound healing. Nonetheless, the number of studies incorporating calcium as a biomolecule in scaffolds designed to accelerate wound healing is still extremely limited.

Calcium can be incorporated into scaffolds prepared using many different techniques as it is very stable and versatile. The regulation of the calcium release from the scaffold in vivo is crucial in order to achieve optimal healing. There is still more to be done to identify the ideal calcium concentrations for different types of wounds at different phases of healing.

In summary, several studies have showed the potential of incorporating and controlling calcium release from scaffolds to accelerate wound healing. However, the number of studies reported thus far is extremely limited. More preclinical and clinical studies are required to validate the findings of these studies.

## Figures and Tables

**Table 1:** Characteristics and indications of wound dressings that are widely used in the clinic.

Type of Dressing	Advantage	Disadvantage	Type of Wound	Commercially Available Product	Reference
Gauze	Cheap	Frequent changes irritate the skin		Curity Vaseline	[26]
Semi-permeable film	Easy wound examination Support autolytic debridement	Rips skin upon removal	Epithelializing wounds Superficial wounds Shallow wounds with low exudate	Opsite Tegadarm	[27]
Semi-permeable foam	High absorbance	Requires frequent changes	Lower leg ulcer Moderately to highly exuding wounds	Lyofoam Allevyn	[28]
Hydrogel	Hydrating Easy application and removal	Easily infected Low mechanical strength	Dry chronic wounds Pressure ulcer Burn	Intrasite Nu-gel	[28]
Hydrocolloid	Support autolytic debridement Absorbs exudate No pain on removal	Not advisable to be used for highly exuding wounds	Lightly to moderately exuding wounds	Granulex Comfeel	[28]
Alginate	High absorbance	Dehydrates wounds	Moderate- to heavy-drainage wounds	Sorban Kaltostat	[29]
Bioactive	Biocompatible Biodegradable Promotes natural healing	Expensive	Heavy-drainage wounds Diabetic ulcers	Grafix Amnioband	[29]
Tissue-engineered skin substitute	Biocompatible Addresses deficient growth factors and cytokines	Expensive Risk of infection Antigenicity	Diabetic ulcers Venous ulcers	Alloderm Integra	[30]

**Table 2:** Studies using calcium-based dressings to promote wound healing.

Study	Key Finding	Reference
Calcium alginate enhances wound healing by upregulating the ratio of collagen types I/III in diabetic rats	Calcium alginate dressing showed excellent cytocompatibility and histocompatibility, and promoted diabetic wound healing by expediting wound re-epithelialization, attenuating inflammatory reactions, and increasing collagen synthesis and wound tensile strength.	[70]
Chitosan-calcium alginate dressing promotes wound healing: A preliminary study	Chitosan-calcium alginate dressing was able to promote wound healing by suppressing the inflammation, promoting angiogenesis, and preserving wound moisture. In addition, the scaffold also possesses antibacterial property and excellent biocompatibility, with no cytotoxicity.	[71]
Preparation and characterization of chitosan/gelatin/nanocrystalline cellulose/calcium peroxide films for potential wound dressing applications	The addition of calcium peroxide particles increased the antibacterial activity of the films against *E. coli*.	[72]
Effective wound healing by antibacterial and bioactive calcium-fluoride-containing composite hydrogel dressings prepared using in-situ preparation	The calcium and fluoride ions released from the hydrogel dressings enhanced the migration of fibroblasts and endothelial cells, as well as inhibiting bacterial growth. In vivo, the ions were found to accelerate wound healing, as indicated by the faster wound re-epithelialization, higher migration of surrounding skin cells, and higher recruitment of inflammatory cells.	[74]

**Table 3:** Studies using calcium-releasing nanoparticles.

Study	Key Finding	Reference
Degradable, antibacterial silver exchanged mesoporous silica spheres for hemorrhage control	Mesoporous silica spheres (MSSs) with calcium are more effective compared to MSSs without calcium in supporting thrombosis and platelet adhesion in vitro, as well as stopping the bleeding in vivo.	[58]
Metal doped calcium silicate biomaterial for skin tissue regeneration in vitro	The zinc-doped calcium silicate particles were non-toxic to the fibroblasts at low concentrations (≤0.1 mg/mL), promoted fibroblast wound closure, and were antibacterial in vitro.	[67]
Wound healing-promoting effects stimulated by extracellular calcium and calcium releasing nanoparticles on dermal fibroblasts	The calcium-phosphate-based ormoglass nanoparticles, coded SG5, and extracellular calcium at similar concentrations were found to increase skin fibroblast migration, collagen synthesis, and cytokine release, as well as decreasing the cell contraction ability. However, lower expression of inflammatory factors and MMP activity were recorded in the SG5 group compared to the extracellular calcium group. These results indicate that SG5 is more suitable to promote the healing of chronic wounds.	[8]
Polymeric composite dressings containing calcium-releasing nanoparticles accelerate wound healing in diabetic mice	The poly(lactic acid) (PLA) nanofiber mats with SG5 nanoparticles promote angiogenesis, collagen synthesis, wound re-epithelialization, and fibroblast migration in diabetic mice with ischemic wounds compared to PLA nanofiber mats and Mepilex^®^.	[75]

**Table 4:** Scaffolds loaded with calcium for skin tissue engineering purposes.

Study	Key Finding	Reference
Allogeneic bilayered tissue-engineered skin promotes full-thickness wound healing in ovine model Full-thickness skin wound healing using autologous keratinocytes and dermal fibroblasts with fibrin: Bilayered versus single-layered substitute	The bilayered skin. consisting of a fibrin-fibroblast layer overlaid with a fibrin-keratinocyte layer, was crosslinked with calcium chloride and it was found to promote wound healing in mice and sheep.	[12,76]
Freeze-gelled alginate/gelatin scaffolds for wound healing applications: An in vitro, in vivo study	Alginate/gelatin hydrogel scaffolds showed biocompatibility and promoted the healing of second-degree burn wounds in vivo, with scaffolds with fibroblasts demonstrating better wound healing properties compared to scaffolds without cells.	[56]
Pectin/carboxymethyl cellulose/microfibrillated cellulose composite scaffolds for tissue engineering	Pectin/carboxymethyl cellulose/microfibrillated cellulose (pectin/CMC/MFC) composite scaffolds were gelated with calcium ions and scaffold with 0.1% (*w*/*v*) MFC demonstrated the highest cell viability.	[68]
Design and evaluation of atorvastatin-loaded chitosan-hydroxyapatite composite bioscaffolds for wound-healing activity	Atorvastatin-loaded chitosan-hydroxyapatite composite scaffolds crosslinked with calcium chloride possessed antibacterial activity and promoted skin wound healing in vivo.	[70]

## Data Availability

Not Applicable.
